# A Unique Acylated Flavonol Glycoside from *Prunus persica* (L.) var. Florida Prince: A New Solid Lipid Nanoparticle Cosmeceutical Formulation for Skincare

**DOI:** 10.3390/antiox10030436

**Published:** 2021-03-12

**Authors:** Eman S. Mostafa, Ahmed Maher, Dalia A. Mostafa, Sameh S. Gad, Mahmoud A.M. Nawwar, Noha Swilam

**Affiliations:** 1 Department of Pharmacognosy, Faculty of Pharmacy, October University of Modern Sciences and Arts (MSA), Giza 12451, Egypt; 2 Department of Biochemistry, Faculty of Pharmacy, October University for Modern Sciences and Arts (MSA), Giza 12451, Egypt; 3 Department of Pharmaceutics, Faculty of Pharmacy, October University of Modern Sciences and Arts (MSA), Giza 12451, Egypt; damostafa@msa.eun.eg; 4 Department of Pharmacology, Faculty of Pharmacy, October University for Modern Sciences and Arts (MSA), Giza 12451, Egypt; sameh.shaaban@msa.eun.eg; 5 National Research Centre, Department of Phytochemistry, Dokki, Cairo 12622, Egypt; ma.el-moien@nrc.sci.eg; 6 Department of Pharmacognosy, Faculty of Pharmacy, The British University in Egypt (BUE), Cairo 11837, Egypt; noha.swilam@bue.edu.eg

**Keywords:** kaempferol 3*-O*-*β*-*^4^C_1_*-(6″-*O*-3,4-dihydroxyphenylacetyl glucopyranoside) by-products, solid lipid nanoparticles, antioxidant, in-vitro skin related enzymes, in-vivo anti-wrinkle

## Abstract

Polyphenols are known dietary antioxidants. They have recently attracted considerable interest in uses to prevent skin aging and hyperpigmentation resulting from solar UV-irradiation. *Prunus persica* (L.) leaves are considered by-products and were reported to have a remarkable antioxidant activity due to their high content of polyphenols. This study aimed at the development of a cosmeceutical anti-aging and skin whitening cream preparation using ethanol leaves extract of *Prunus persica* (L.) (PPEE) loaded in solid lipid nanoparticles (SLNs) to enhance the skin delivery. Chemical investigation of PPEE showed significantly high total phenolic and flavonoids content with notable antioxidant activities (DPPH, ABTS, and *β*-carotene assays). A unique acylated kaempferol glycoside with a rare structure, kaempferol 3*-O-β*-*^4^C_1_*-(6″-*O*-3,4-dihydroxyphenylacetyl glucopyranoside) (KDPAG) was isolated for the first time and its structure fully elucidated. It represents the first example of acylation with 3,4-dihydroxyphenyl acetic acid in flavonoid chemistry. The in-vitro cytotoxicity studies against a human keratinocytes cell line revealed the non-toxicity of PPEE and PPEE-SLNs. Moreover, PPEE, PPEE-SLNs, and KDPAG showed good anti-elastase activity, comparable to that of *N*-(Methoxysuccinyl)-Ala-Ala-Pro-Val-chloromethyl ketone. Besides, PPEE-SLNs and KDPAG showed significantly (*p* < 0.001) higher anti-collagenase and anti-tyrosinase activities in comparison to EDTA and kojic acid, respectively. Different PPEE-SLNs cream formulae (2% and 5%) were evaluated for possible anti-wrinkle activity against UV-induced photoaging in a mouse model using a wrinkle scoring method and were shown to offer a highly significant protective effect against UV, as evidenced by tissue biomarkers (SOD) and histopathological studies. Thus, the current study demonstrates that *Prunus persica* leaf by-products provide an interesting, valuable resource for natural cosmetic ingredients. This provides related data for further studying the potential safe use of PPEE-SLNs in topical anti-aging cosmetic formulations with enhanced skin permeation properties.

## 1. Introduction

Skin aging is a complex multifactorial progressive process that catalyzes physical changes in skin and connective tissue [1]. It is classified into intrinsic and extrinsic aging. Extrinsic skin aging results mainly from environmental factors, such as pollutants, smoking, life-stress, or predominantly by repeated exposure to UV radiation (photoaging) [2].

Overexposure to UV radiation stimulates the overproduction of reactive oxygen species (ROS) which results in endogenous oxidative stress in skin tissues leading to degradation of the extracellular matrix (ECM) components. ECM degradation is directly linked to skin aging and is responsible for the increase in activity of certain enzymes such as collagenase, elastase, and tyrosinase that are involved in skin aging. These dermal enzyme activations cause a decrease in the levels of elastin, and collagen which leads to loss of elasticity and strength of skin and the appearance of wrinkles [3]. Also, induction of excessive melanin production and skin tanning results in hyperpigmentation of the skin.

Therefore, skin aging can be prevented by antioxidants with free radical scavenging activity which may have a great significance in the defense and therapeutics of age-related diseases involving ROS [4]. The other way to retard aging is using elastase, collagenase, and tyrosinase inhibitors which are very strong candidates for anti-wrinkle and skin whitening activities endorsing the preservation of skin elasticity and can be a common approach to deal with pigmentation disorders [5].

It has been reported that natural antioxidants isolated from plants attenuate the risk of photoaging induced by UV irradiation both in-vitro and in-vivo [6] and they are more preferable as cosmeceutical ingredients than synthetic antioxidants in respect of cost and side effects. In addition, many in-vitro and in-vivo studies have shown that phenolic compounds such as phenolic acids, flavonoids, and tannins could scavenge free radicals and inhibit elastase, collagenase, and tyrosinase enzymes [7].

The use of natural antioxidant in topical preparations to protect skin against oxidative stress caused by extrinsic factors have been reported [8]. However, the majority of antioxidant molecules are innately unstable and can easily oxidize to form inactive compounds before reaching the site of action which makes them difficult to formulate in an appropriate and stable cosmetic product. These findings prove the necessity of unique delivery systems to enhance these antioxidant formulations [9].

Several factors affect the permeability of topically applied polyphenols; the phenolic subclass, its structure, its molecular size, and the glycoside or aglycone form, as well as the other formulation components. However, the interaction with skin components through a mixture of physical and chemical methods could improve permeability through a reversible disruption of the stratum corneum layer’s barrier structure. Encapsulation approaches is one of the permeability enhancing methods most commonly used to easily stabilize polyphenolics during storage, increasing their antioxidant effects, dermal absorption, and penetration in cosmetic and topical therapies. Newly developed encapsulation technologies include nanoemulsions, transferosomes, solid lipid nanoparticles, nanocrystals, and cubosomes [10].

Solid lipid nanoparticles (SLNs) are a stable delivery system for skin products. SLNs are considered promising drug carriers for topical formulations due to their photostability, controlled release properties, occlusivity, odor masking effects, and penetration enhancement of the active constituents through the skin by increasing its hydration with no signs of skin irritation, in addition to simplicity of production, low toxicity, and physical stability [9,11].

Therefore, it is highly important to look for a relevant source of plant polyphenols as possible therapeutic anti-wrinkle and skin whitening agents using nanocarriers delivery system, along with its ability to improve the body’s antioxidant system.

On a worldwide basis, there are about 2000 peach cultivars [12]. *Prunus* (Rosaceae) is a famous plant genus that includes phenolic-rich species [13,14,15]. Some species of this genus are cultivated in Egypt for their edible fruits or as ornamentals [16].

*Prunus persica* (L.) Batch (PP) is a small tree with glabrous twigs and a short trunk, a spreading, rounded crown [12] and commonly cultivated in West Asia, Europe, India, and North Africa [17]. Traditionally, its leaves have been used as diuretics, laxatives, vermifuges, insecticides, sedatives, for whooping cough, for the treatment of leukoderma, and as febrifuges [17]. Furthermore, pharmacological studies on leaves characterized their in-vivo anti-diabetic, spasmogenic effect, anti-inflammatory, anticoagulant, hepatoprotective, antimalarial, anti-asthmatic, and in-vitro cytotoxic, antimicrobial, and nitric oxide inhibitory with significant anti-oxidant activities [18,19,20,21,22].

Many studies have been done to investigate the phenolic profile of PP flowers, fruits, and seeds, their antioxidant activities, pharmacological and nutritional values [13,14,23,24]. However, very scarce data were available on the leaves. To date, a single report is available describing the isolation of five flavonol glycosides from an ethanol leaves extract [25]. In addition, phenolic compounds have been characterized, including phenolic acids and flavonoids using HPLC-MS analysis with flavonols as dominant substances among all the determined compounds [20,26].

Moreover, although PP seeds, fruits, flowers, and other species have been reported to have in-vivo protective activity against UV-induced photoaging and in-vitro anti-wrinkle and skin whitening activates [15,23,27,28,29,30,31,32], this has never been reported for PP leaves extract.

Large amounts of PP leaves are by-products derived from peach tree cultivation and the fruit canning industries [33]. The by-products of PP seeds, fruits, and other leaf varieties have been evaluated in cosmetics and as dietary food for potential valorization [32,34,35]. However, to the best of our knowledge, the leaves by-products of PP var. Florida Prince have never been investigated and could be an interesting source of phytochemicals. Compared with other industries, the cosmetic market is more accessible and expanding and can be a source for valorization of by-products [15]. Actually, skin care and skin aging products are some of the most important cosmetic potentials.

In a continuation of our research on polyphenolics of Egyptian edible plants [36] and since PP leaves are considered by-products and were reported to have significant antioxidant activity mainly due to their high flavonols content, this study aims to subject PPEE to an extensive phytochemical analysis of its phenolic profile and to incorporate PPEE into loaded SLNs as a unique skin delivery system. Furthermore, the anti-aging and skin whitening cosmetic potential were evaluated by investigating the in-vitro antioxidant activity of PPEE, PPEE-SLNs, and the isolated constituents, along with the evaluation of their inhibitory effects against skin-related enzymes. In addition to incorporation of PPEE-SLNs into a topical cosmeceutical anti-aging cream and to test the product safety as well as evaluation of the possible in-vivo anti-wrinkle activity of PPEE-SLNs cream formulations against UV-induced photoaging in a mouse model. This could provide data for further studying the potential use of PPEE-SLNs leaves in topical formulations with enhanced skin permeation.

## 2. Material and Methods

### 2.1. General

NMR spectra were acquired in DMSO-*d_6_* on an Avance 400 NMR spectrometer (Bruker biospin Gmbh, Rheinstetten, Germany) at 400 MHz. Standard pulse sequence and parameters were used to obtain one-dimensional ^1^H and ^13^C-APT, and two-dimensional HSQC and HMBC spectra. ^1^H chemical shifts (δ) were measured in ppm, relative to TMS and ^13^C-NMR chemical shifts to DMSO-*d_6_* and were converted to TMS scale by adding 39.49. ORAC measurements were performed on a FLUOstar Omega Microplate Reader (BMG LABTECH Gmbh, Ortenberg, Germany). A Shimadzu UV–Visible-1601 spectrophotometer (Shimadzu, Kyoto, Japan) was used for recording the UV spectra. Chromatographic analysis (PC) was carried out on Whatman No. 1 paper, using solvent systems: (1) H_2_O; (2) 6% HOAc; (3) BAW (*n*-BuOH–HOAc–H_2_O, 4:1:5, upper layer). For 2-DPC, solvent 2 was used for the first way and solvent 3 for the second way. Materials: chemicals, solvents, elastase, collagenase, tyrosinase enzymes, and reference drugs (EDTA, kojic acid, and *N*-(Methoxysuccinyl)-Ala-Ala-Pro-Val-chloromethyl ketone with purities >95%) were obtained from Sigma-Aldrich (Sigma-Aldrich, MO, USA). 

### 2.2. Plant Materials

Leaves of *Prunus persica* (L.) Batsch var. Florida Prince were collected in June 2020 from EL-Hashash Farm, El-Bastan entrance, Cairo-Alexandria desert road K. 103, 18 k. inside, Egypt. The voucher specimen (4643) was placed in Herbarium of Flora and Phytotaxonomy Researches Department (CAIM), Horticultural Research Institute, Agricultural Research Center, Giza, Egypt. Prof. Dr. Abdel-Haleem Abdel-Mogaly, Prof. of Botany at Agricultural Research Centre, has confirmed its identity.

### 2.3. Preparation of Plant Extract

Extraction of PP leaves sample (2 kg) was done using hot EtOH/H_2_O (3:1, three times, 3 L each, for 8 h, under reflux) followed by solvent evaporation under reduced pressure at 50 °C yielding an amorphous extract, PPEE, with brownish dark color (101 g).

### 2.4. Determination of Total Polyphenols Content (TPC)

The Folin–Ciocalteu reagent as described by Li et al., [37] was used to estimate the amount of total phenolic content in PPEE, presented as mg equivalent gallic acid/g dry extract.

### 2.5. Determination of Total Flavonoid Content (TFC)

The aluminum chloride colorimetric method described by Bahromun et al., [38] was used to estimate the total flavonoid content. The results are presented as mg equivalent quercetin/g dry extract.

### 2.6. Fractionation of PPEE and Isolation of Compound ***1***

The resulting dry material (101 g), dissolved in 150 mL MeOH, was applied to a MCI gel column (CHP20P, 75–150 mm; Mitsubishi Chemical Co., Düsseldorf, Germany) and eluted by MeOH/H_2_O mixtures of decreasing polarities. This results in four major fractions (I–IV). Elution of fraction I was done using 30% MeOH/H_2_O, 22 g; fraction II eluted with 50% aqueous MeOH, 3.1 g; fraction III, with 70% aqueous MeOH (4.6 g); fraction IV, eluted with 90% aqueous MeOH (1.9 g). A concentrated solution of fraction III left standing overnight at room temperature, giving a crude sample of compound **1** (765 mg). This is followed by filtration and re-dissolving in boiling EtOH, then left to stand at room temperature to afford a brownish-yellow pure sample of **1** (530 mg).

### 2.7. Identification of kaempferol 3-O-β-^4^C_1_-(6”-O-3,4-dihydroxyphenylacetyl glucopyranoside) (***1***)

R _f_ values: 34 (H_2_O), 39 (15% HOAc), 44 (BAW). UV in MeOH λ_max_ nm: 268, 316, 368; NaOAc: 267, 317, 369; NaOAc + H_3_BO_3_: 268, 318, 340; NaOMe: 277, 370. Normal acid hydrolysis gave glucose (Co-PC), kaempferol (Co-PC), and 3,4-dihydroxyphenyl acetic acid (UV and ^1^H NMR spectra), UV λ_max_ (MeOH): 224, 282 nm; λ_max_ (MeOH + A1C1_3_): 220, 250 (sh), 291 nm; (MeOH + NaOAc + H_3_BO_3_) 211, 234 (sh), 288 nm. ^1^H NMR: δ ppm: 3.40 (s, 2H, C-H_2_), 6.60 (m, 3H aromatic, H-2, H-5, H-6), *β*–glucosidase treatment of **1** [lyophilized, chromatographically pure, salt-free enzyme from almond, BDH Merck, Poole Dorset, UK (E.C. 3.2.1.21)] for 24 h, at 37 °C in acetate buffer, pH 5.1: the compound was recovered unchanged. ^1^H-NMR of **1**: δppm: kaempferol moiety: 8.0.3 (d, *J* = 8 Hz, H-2′ and H-6′), 6.89 (d, *J* = 8 Hz, H-3′ and H-5′), 6.45 (d, *J* = 2 Hz, H-6), 6.21 (d, *J* = 2 Hz, H-8); glucose moiety: 5.42 (d, *J* = 8 Hz, anomeric proton H-1″), 4.04 (dd, *J* = 12 Hz and *J* = 4.5 Hz, H-6″a), 4.2 (d, *J* = 12 Hz, H-6″b); 3,4-dihydroxyphenyl acetic acid moiety: 3.46 (broad singlet, Δν_1/2_ = 4 Hz, CH_2_-7), 6.67 (m, 3H, H-2‴, H-5‴, H-6‴). ^13^C-NMR of **1**: δ ppm: kaempferol moiety:156.65 (C-2), 133.5 (C-3), 178.01 (C-4), 161.69 (C-5), 99.13 (C-6), 164.58 (C-7), 93.90 (C-8), 156.71 (C-9), 104.39 (C-10), 121.34 (C-1′), 133.66 (C-2′ and C-6′), 161.63 (C-4′), 115.20 (C-3′ and C-5′); glucose moiety: 99.13 (C-1″), 73.85 (C-2″), 76.88 (C-3″),70.35 (C-4″), 76.96 (C-5″), 62.25 (C-6″); 3,4-dihydroxyphenyl acetic acid moiety: 127.87 (C-1‴), 116.35 (C-2‴), 145.33 (C-3‴), 148.92 (C-4‴), 121.57 (C-5‴), 117.0 (C-6‴), 176.26 (C=O), 40.02 (C-H_2_).

### 2.8. In-Vitro Studies

#### 2.8.1. Evaluation of Cytotoxicity in Human Keratinocytes

The human keratinocytes cell line was obtained from VACSERA CO. (The Egyptian Company for the Production of Vaccines, Sera, and Drugs, Giza, Egypt). The MTT assay was done for PPEE and PPEE-SLNs according to the method of Mostafa et al. [36].

#### 2.8.2. In-Vitro Antioxidant Assays

The DPPH assay was peformed using the method of Yardpiroon et al. [39], Vitamin C was used as a positive control. The ABTS assay followed the method of Re et al., [40], Vitamin C was used as a standard. The β-carotene bleaching assay used the method described by Soulef et al., [14] in the presence of the positive control BHT. The value for each test sample was presented as the inhibition curve at 50% or IC_50_.

#### 2.8.3. Estimation of the Anti-Elastase, Anti-Collagenase, and Anti-Tyrosinase Activities

The anti-aging and skin whitening activities were assessed following the methods reported by Mostafa et al. [41]. For the anti-elastase assay, 1 μg/mL of human leukocyte elastase was incubated with HEPES buffer pH 7.5 and each of the tested extracts or 1.4 mg/mL N-Methoxysuccinyl-Ala-Ala-Pro-chloromethyl ketone (standard inhibitor) in a 96-well plate for 20 min at room temperature before the addition of 100 μL of 1 mM *N*-Methoxysuccinyl-Ala-Ala-Pro-Val-*p*-nitroanilide as a substrate. After 40 min incubation the absorbance was measured at 405 nm with a well devoid of extract/inhibitor serving as a blank. The tested samples were used in a concentration range of 25–300 µg/mL.

For anti-collagenase, 1 mg/mL of collagenase type 1 from *Clostridium histolyticum,* its buffer (pH 7.4) and each of the tested extracts or 1.4 mg/mL EDTA (standard inhibitor) were incubated for 20 min at 37 °C before adding 100 μL of FALGPA as a substrate. The mixture was incubated for another 1 h at 37 °C before adding 200 μL 2% ninhydrin in 200 mM citrate buffer (pH 5) and putting in a boiling water bath for 5 min. The solution was then allowed to cool. 200 μL of 50% isopropanol was then added and the absorbances measured at 540 nm. The tested samples were used in a concentration range of 25–300 µg/mL.

For the anti-tyrosinase assay, 5600 units/mL of mushroom tyrosinase was incubated with each of the tested extracts or 1.4 mg/mL kojic acid (standard inhibitor) for 15 min at 37 °C before adding 1 mM L-DOPA as a substrate. The absorbance of the formed dopachrome was measured at 475 nm. The tested samples were used in a concentration range of 75–500 µg/mL.

### 2.9. Formulation and Characterization of PPEE-SLNs

The formulation was done according to the method of Choubey et al. [11]. First, glyceryl monostearate was dissolved in a chloroform–methanol mixture (1:1) and then the ethanolic extract was dispersed in that solution. Later the organic solvents were removed using a rotovap and the solution heated. Tween 80 was then added and the mixture stirred at 3000 rpm for 30 min then homogenized for 4 h before filtering and drying. Characterization of PPEE-SLNs was examined by estimating shape and surface morphology, particle size, polydispersity index (PDI), zeta potential analysis, percent entrapment efficiency (PEE), and Fourier transform infrared (FT-IR) spectroscopy studies.

### 2.10. Preparation of PPEE Topical Cream Formulae

#### 2.10.1. Formulation of PPEE-SLNs Cream

Two formulations of cream (2% & 5% of PPEE-SLNs) were prepared as shown in Table 1 according to the method of Mahawar et al. [42].

#### 2.10.2. Evaluation Parameters of PPEE-SLNs Cream

Organoleptic properties, pH of the cream, spreadability studies, viscosity test, homogeneity test, patch test (Burchard test), in-vitro skin permeation examination, and stability studies (ICH guidelines) were evaluated according to Matangi et al. [43]. A microbial limit test was carried out according to Sekar et al. [44].

### 2.11. In-Vivo Anti-Wrinkle Study of PPEE-SLNs Cream

#### 2.11.1. Experimental Animals

Fifty male hairless mice (HR-1) (4-week-old), 17–24 g were obtained from VACSERA. The experiment was carried out after the approval of the ethics committee at October University for Modern Sciences and Arts (MSA), Protocol number (PG1/EC1/2020PD) sample size determined according to (G. Power) software. One week before the in-vivo anti-wrinkle study, mice were freely accessed to food, water and become acclimatized to the air-conditioned room (23 ± 2 °C). Mice (50) were divided into five groups, randomly (*n* = 10) as shown in Table 2. Treatment was done over 40 days, three times weekly, with 0.5 g of PPEE-SLNs cream or any other treatment and applied to the dorsal mice skin and subsequently exposed to UV (365 nm).

#### 2.11.2. Wrinkle Score Measurement

Before and after topical treatments, two times/week, dorsal mice skin photos were taken. The skin wrinkle grading was evaluated using Bissett’s visual wrinkle scale [45]. The bases for the grading scale were the texture of wrinkle (fine or coarse) and keratosis, dividing into five grades: worse (−2), slightly worse (−1), no change (0), slightly improved (+1), and improved (+2).

#### 2.11.3. Histological Evaluation

Histological evaluation was done according to the method of Elder et al. [46].

#### 2.11.4. Superoxide Dismutase (SOD) Activity

SOD activity was estimated according to the method of Ukeda et al. [47].

### 2.12. Statistical Analysis

All samples were analyzed in triplicates. Data were represented as means ± standard deviation (SD). The IC_50_ values (concentration required for 50% inhibition) were calculated by making a linear regression curve showing sample concentrations and percentage inhibition. Statistical comparison was done using one-way analysis of variance (ANOVA) completed by Tukey’s test for multiple comparisons. Different values of antioxidant activities and enzyme inhibition assays were compared using the Pearson correlation test and differences were considered statistically significant at *p* < 0.05. GraphPad Prism 5.03 for Windows (GraphPad Software, San Diego, CA, USA) was used to perform these analyses.

## 3. Results

### 3.1. Total Phenolic and Flavonoid Contents

TPC was determined spectrophotometrically in PPEE as 387.5 ± 4.28 mg GAE/g extract. Also, TFC was estimated as 241.7 ± 3.25 mg QE/g extract. PPEE showed high concentrations of phenolic and flavonoid compounds. Both results are following values previously reported for PP leaf extracts confirming that flavonoids are the main chemical constituents in PP leaves [20].

### 3.2. Isolation and Structure Elucidation of kaempferol 3-O-β-^4^C_1_-(6″-O-3,4-dihydroxyphenylacetyl glucopyranoside), Compound ***1***

After sorting out all the known structures in that extract, the search for unique, potentially biologically active compounds became more efficient. According to the received 2-DPC analytical data the phenolics of PPEE were found to be best fractionated over an MCI gel column eluted by MeOH/H_2_O mixtures of decreasing polarities, a process which afforded four major column fractions. An amorphous material was separated from a hot concentrate of fraction III (desorbed from the column by 70% aqueous MeOH) on standing overnight at room temperature. Crystallization of this material, twice from boiling EtOH yielded a chromatographically pure brownish-yellow amorphous powder **1**. It showed chromatographic properties (dark purple spot-on PC under UV light, turning lemon yellow when fumed with ammonia vapor with moderate migration in aqueous and organic solvents) and color reactions (a lemon-yellow color with Naturstoff reagent), which suggested a kaempferol derivative bearing a free 4′ hydroxyl group and an *O*-substituted 3-*O*-position. The UV spectra of **1** in MeOH (268 nm, 316 nm, 360 nm) and upon addition of the diagnostic shift reagents [48] were typical of those of 3-*O*-glycosylated kaempferol (positive shift with NaOAc, stable shift with NaOMe). Normal acid hydrolysis of **1** (2 N aqueous HCl, 3 h, 100 °C) yielded glucose (comparative paper chromatography, Co-PC), kaempferol, and 3,4-dihydroxyphenyl acetic acid (UV absorption and ^1^H-NMR spectra, for individual samples, isolated by preparative paper chromatograms of the ethyl acetate extract of the hydrolysate). Consequently, **1** is kaempferol 3-*O*-(3,4-dihydroxyphenyl acetyl glucoside). The compound is recovered unchanged after being incubated with *β*-glucosidase enzyme for 24 h, thus proving that the glucosyl moiety is acylated. The molecular formula of **1** was concluded to be C_29_H_26_O_14_ from its negative HRESI mass spectrum which showed an [M-H]^−^ ion at *m/z* = 597.5302 (calc. for C_29_H_25_O_14_, 597.5015). The ESI-MS experiment (negative-ion mode) gave a quasi-molecular ion peak [M-H]^−^ at *m*/*z*: 597 indicating a molecular weight of 598 for **1**. Further fragment ion peaks in the ESI-MS-MS spectrum were observed at *m*/*z*: [M-H]^−^: 447, 167, and 285 corresponding to the loss of dihydroxyphenyl acetate from the parent compound **1** while the fragment ions at *m*/*z* 285 and *m*/*z* 167 were attributed to the kaempferol and dihydroxyphenylacetic acid moieties, respectively. To determine the site of attachment of all moieties in the molecule of **1** and to allow the full assignment of all carbon and proton resonances, NMR spectroscopic analysis of **1**, including 1D- ^1^H and ^13^C, and 2D- HSQC and HMBC, was then carried out. From the five signals in the sugar region between δ ppm 62.25 and 76.9 and from the anomeric carbon signal located at 99.13 ppm it was proved that the sugar moiety must be attached to position 3 of kaempferol because this C-3 carbon signal was shifted upfield and the corresponding *ortho* and *para*-carbon signals were shifted downfield (see experimental). Similar shifts are well-known from the work of Nawwar et al. [48]. This was further confirmed by the *^3^J long*-range correlations recognized in the HMBC spectrum, whereby, one cross signal was found correlating the anomeric glucose proton H-1″ signal at δ 5.42 to the flavonol C-3 carbon signal at δ 133.5. The *β-*configuration of the glucose moiety was derived from the C-l″ chemical shift at 99.13 ppm. The chemical shift values of the sugar carbons confirmed the pyranose form of this moiety [48]. The ^1^H-NMR spectrum of **1** was also in accordance with the proposed structure. The chemical shift of the anomeric proton signal at δ 5.42 ppm (d, *J* = 8 Hz) indicated that the anomeric carbon is attached to the kaempferol moiety at C-3 (δppm 133.5) and the determined coupling constant 8 Hz, prove the *β*-configuration of the glucose moiety. The conformation of the sugar moiety is *^4^C_1_* as followed from the *β*-configurations discussed above.

**Figure 1:**
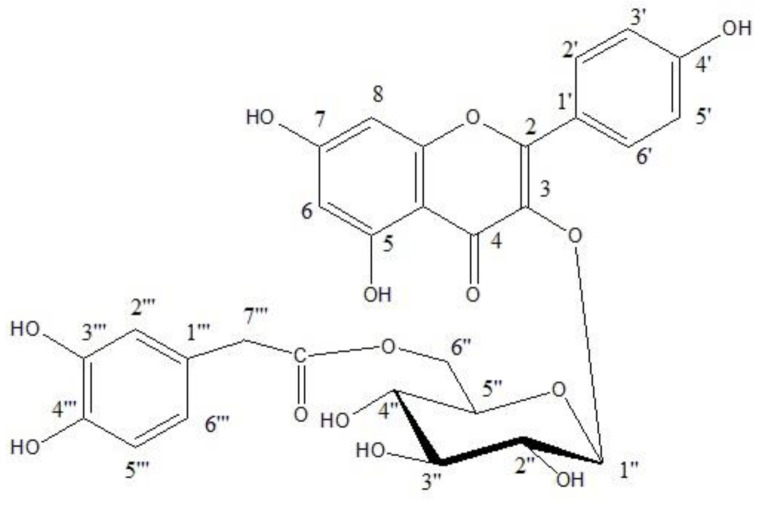
kaempferol 3-*O-β-^4^C_1_*-(6″-*O*-3,4-dihydroxyphenylacetyl glucopyranoside).

Also, attachment of the 3,4-dihydroxyphenylactetic acid moiety to the C-6″ methylene glucopyranose moiety followed from the downfield shift of the signal of this carbon to δ ppm 62.25 in the ^13^C-NMR spectrum. This was further confirmed by the cross peak in the HMBC spectrum, correlating the methylenic glucose protons signals at δ 4.04 (H-6″a) and δ 4.2 ppm (H-6″b) to the carbonyl carbon of the 3,4-dihroxyphenylacetic acid moiety at δ 176.26 ppm. These and the above-given data finally confirmed the structure of compound **1** to be the new kaempferol 3*-O*-β-^4^C_1_-(6″-*O*-3,4-dihydroxyphenylacetyl glucopyranoside) (KDPAG), reported for the first time in nature, as it represents the first acylation with 3,4-dihydrocyphenyl acetic acid in association with flavonoid chemistry (Figure 1).

### 3.3. In-Vitro Studies

#### 3.3.1. Evaluation of Cytotoxicity in Human Keratinocytes

**Figure 2:**
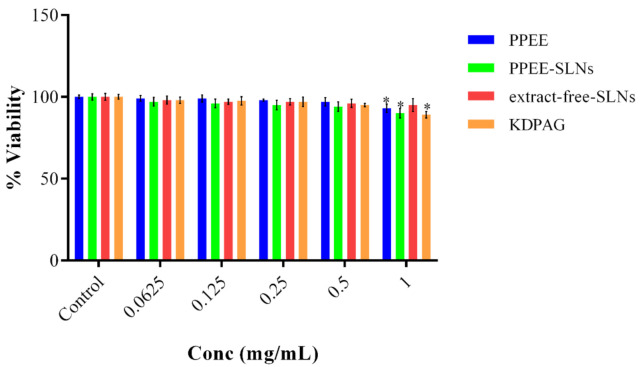
Viability of human keratinocytes treated with PPEE, PPEE-SLNs, Extract-free-SLNs, and KDPAG for 24 h; At the concentration of 1 mg/mL, there was a significant reduction in the viability of the cells treated with PPEE, PPEE-SLNs and KDPAG. The data represents% cell viability at each concentration (mean ± S.D.), *n* = 3. Statistical significance using One-way ANOVA, followed by Tukey’s test: * *p* < 0.05**.**

The MTT assay was used to test whether PPEE, PPEE-SLNs, and KDPAG could affect cell viability using human keratinocytes. Different concentrations of PPEE, PPEE-SLNs, extract-free-SLNs and KDPAG at 0.0625– 1mg/mL were incubated over 24 h with a human keratinocytes cell line (Figure 2). Over 24 h, there was no observed significant reduction in human keratinocytes cell viability in the concentration range of 0.0625–0.5 mg/mL (*p* < 0.05). However, after treatment with 1 mg/mL of PPEE, PPEE-SLNS, Extract-free-SLNs, and KDPAG over 24 h, a significant reduction in cell viability of human keratinocytes was observed, 93%, 90%, 95%, and 89% of the cells survived, respectively. Thus, concentrations of PPEE, PPEE-SLNs, and KDPAG equal to or lower than 0.5 mg/mL were used for subsequent studies.

#### 3.3.2. In-Vitro Antioxidant Assays

The antioxidant activity was evaluated using DPPH, ABTS systems, and *β*-carotene bleaching test. In all tested samples, a concentration-dependent manner was found. In the DPPH assay, KDPAG showed the highest scavenging activity followed by PPEE-SLNs then PPEE. The IC_50_ values were 6.35 ± 3.40 µg/mL, 8.79 ± 2.70 µg/mL, and 10.5 ± 1.81 µg/mL respectively compared to vitamin C used as a standard with an IC_50_ of 2 ± 0.01 µg/mL. This trend was observed also against ABTS radicals with a value of 3.91 ± 1.43 µg/mL, 4.29 ± 1.12 µg/mL, and 6.10 ± 0.62 µg/mL, respectively, compared with the standard Vit. C with an IC_50_ of 0.96 ± 0.02 µg/mL confirming the results of the DPPH assay. Such scavenging activity for PP leaf extracts and their fractions has already been pointed out for both assays but with higher values than the obtained ones [14,49]. These findings suggest that the activity of antioxidants is affected by environmental conditions, plant parts, stage of maturity, method of harvesting, and solvents used for extraction. These results are consistent with the literature as PP leaf extract was found to have a better antioxidant capacity than seed, peel, pulp, and fruit extracts in both assays [14,20,50]. Also, the reported high flavonoidal content of PP leaves, in particular, flavonols was thought to be responsible for the antioxidant activity [20].

As for the other antioxidant assays about peach leaf extract, data are very rare. After 30 min in *β*-carotene assay, IC_50_ values were 1.73 ± 0.05 µg/mL, 1.93 ± 0.25 µg/mL, and 2.03 ± 0.05 µg/mL respectively while the IC_50_ of BHT (the positive control), was 8.06 ± 0.67 µg/mL.

#### 3.3.3. Estimation of the Anti-Elastase, Anti-Collagenase, and Anti-Tyrosinase Activities

PPEE, PPEE-SLNs, and KDPAG showed anti-elastase, anti-collagenase and anti-tyrosinase activity with a high% inhibition at 300 μg/mL and relatively low IC_50_ values (Table 3) compared to their respective positive controls.

### 3.4. Evaluation of PPEE-SLNs and PPEE-SLNs Cream

**Figure 3:**
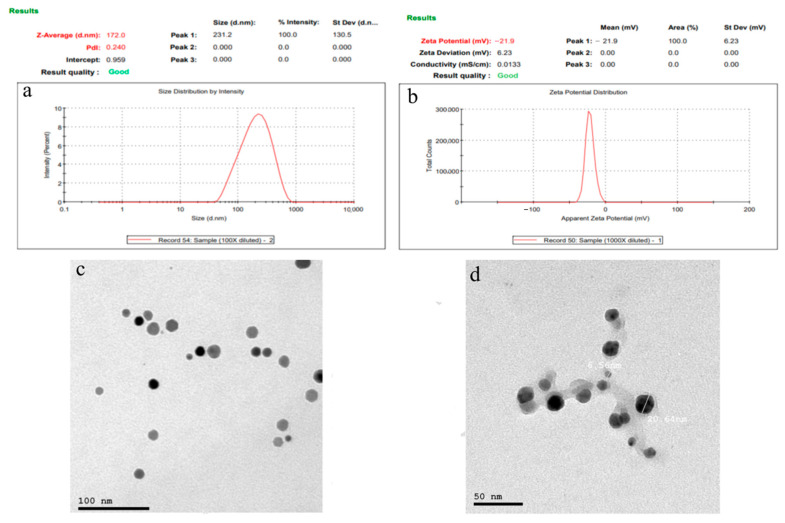
(**a**) Particle size (nm), (**b**) zeta potential (mV), (**c**) Scanning electron microscopy (SEM) (nm) and (**d**) Transmission electron microscopy (TEM) (nm) micrographs of PPEE-SLNs.

**Figure 4:**
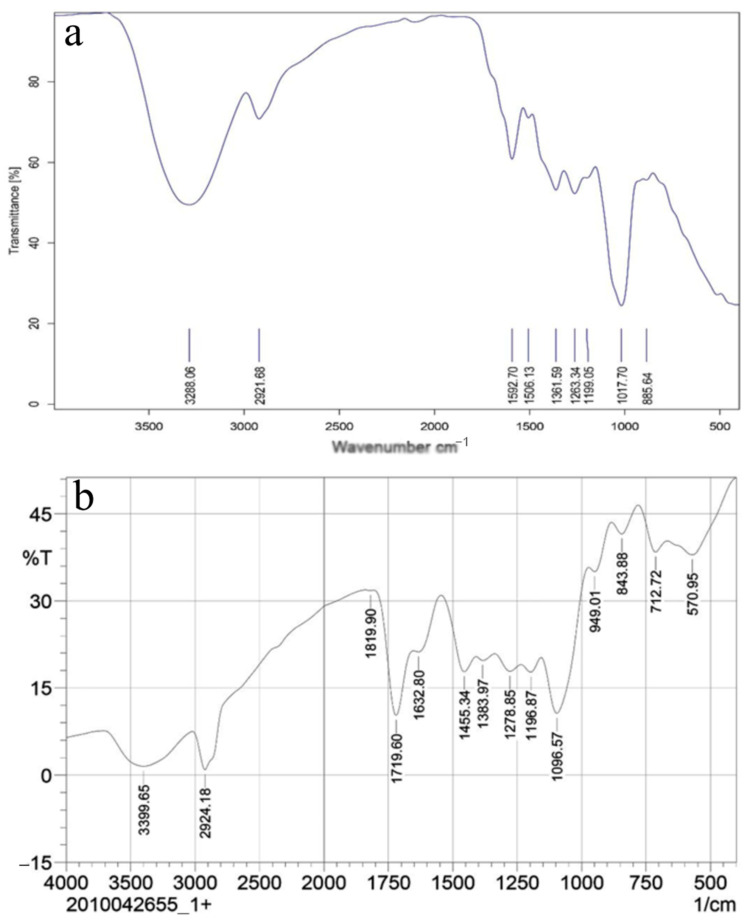
FT-IR of (**a**): PPEE, (**b**) PPEE-SLNs with similar characteristic peaks indicating no significant interaction between PPEE and the lipid.

**Figure 5:**
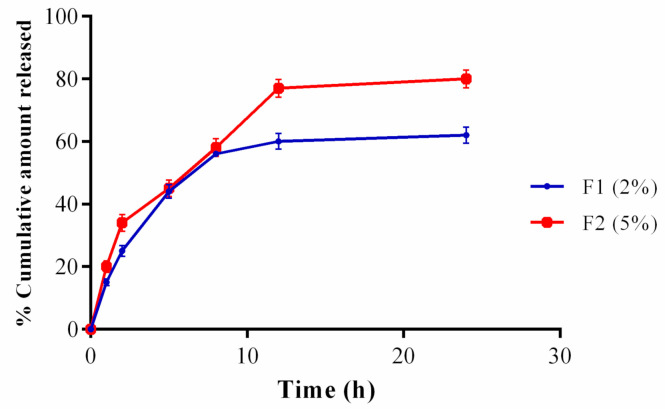
In-vitro permeation study of PPEE cream 2% and 5% Cumulative amount of PPEE released from PPEE-SLNs cream (2% and 5%) is expressed as mean ± S.D.

The prepared PPEE-SLNs formulations showed particle sizes of 170 nm to 176 nm; this indicates that the addition of surfactant to solid lipid nanoparticle systems causes the interfacial film to condense and stabilize. Formulations show low values of polydispersity index (0.230–0.450) indicating homogeneity of particle size distribution with ZP between −21.8 and −22.0 ensuring high stability products (Figure 3a,b). The recorded 70–77% of PPEE-SLNs encapsulation efficacy is due to the type of lipid (glycerol monostearate) which is capable of closing the surface pores in the beads. TEM & SEM micrographs revealed the formation of nanoparticles of narrow particle size distribution, smooth, spherical, and homogenous nanovesicles (Figure 3c,d). In FTIR, PPEE exhibits characteristic peaks at 3352 cm^−1^ corresponding to the aromatic secondary amine N-H stretching, 2974.23 cm^−1^ corresponding to aromatic C-H stretching, 1735.93 cm^−1^ corresponding to C=O stretching and 1257.59 cm^−1^ corresponding to C-N aliphatic amine stretching as appeared in (Figure 4). The FTIR spectra of PPEE and lipid physical mixture exhibit the same characteristic peaks due to the aromatic secondary amine N-H stretching at 3348.42 cm^−1,^ C=O stretching at 1735.93 cm^−1^, and C-N aliphatic amine stretching at 1257.59 cm^−1^. Thus, it is evident that all the characteristic peaks that were present in the spectra of PPEE replicated almost in the same region in the spectra of PPEE-SLNs physical mixture indicating that there is no significant interaction between the drugs and the lipids. PPEE-SLNs creams (2% and 5%) were yellowish with a smooth appearance, smooth surface, and a suitable pH ranging from 5–5.8 ± 0.15 which confirms the compatibility of the formulations with skin secretions, not irritant to human skin, no presence of redness or edema, easily washable with water and good spreadability value ranged from 10–13 g cm/s, with viscosity in the range of 500 ± 6.24 to 600 ± 7.52 CPS at 10 rpm, no evidence of phase separation and good consistency during the study period. The total amount of bacteria and molds was less than 100 CFU/mL and in the acceptance range for skincare products. The 2% cream showed an initial burst release of PPEE-SLNs of about 15.21 ± 1.44% during the first 1 h; following that, the PPEE-SLNs entrapped into the cream were released gradually; 60.32 ± 2.54% were released after 12 and 62 ± 1.44% almost after 24 h respectively. On the other hand, the 5% cream showed an initial burst release of PPEE-SLNs of about 20.21 ± 2.70% during the first 1 h; following that, the PPEE-SLNs entrapped into the cream was released gradually, 77.12 ± 2.88% were released after 12 and 80 ± 2.91% almost after 24 h respectively. Both cream formulae showed extended-release of PPEE over 24 h (Figure 5). Moreover, our research study presented that formulations of cream (2% and 5%) are stable for two months.

### 3.5. In-Vivo Studies

#### 3.5.1. Anti-Wrinkle Activity Exerted by PPEE-SLNs Cream

**Figure 6:**
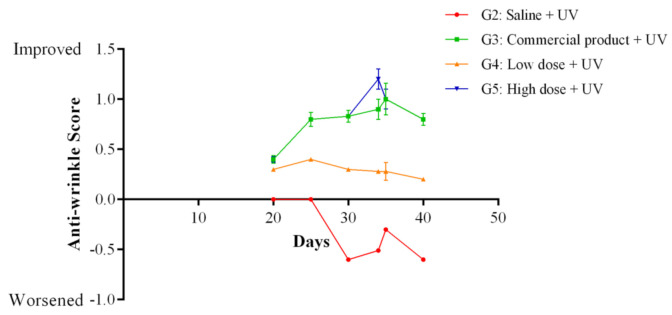
Anti-wrinkle score measurement. The commercial product and the PPEE-SLNs formulations showed a statistically significant increase in the anti-wrinkle activity compared to the control group (G2). 5% PPEE-SLNs (G5) showed comparable activity to the commercial product (G3). *p* < 0.05 / *n*= 10.

**Figure 7:**
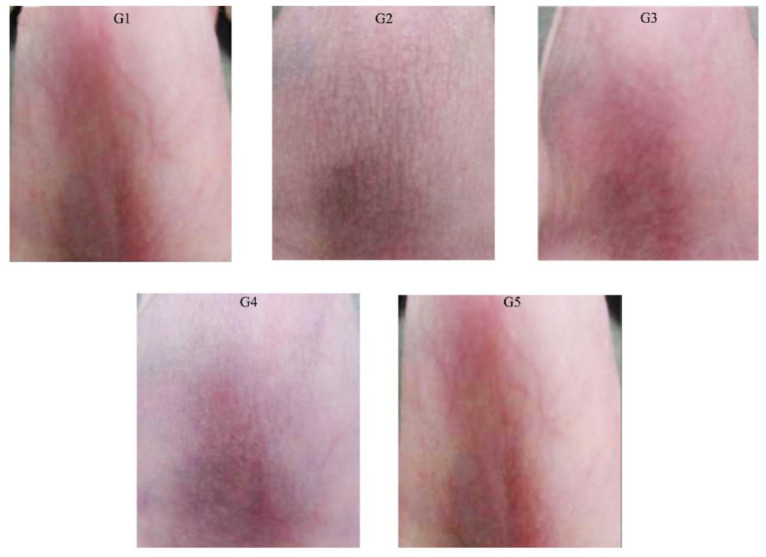
Photographs of hairless mice skins. G1 showed normal skin surface. Thick and deep wrinkles were identified in **G2** whereas **G3**–**G5** showed a very smooth skin surface due to the applied treatment.

After twenty days of treatment with the cream, the anti-wrinkle scores were assessed. The anti-wrinkle effect of PPEE-SLNs cream was dose-dependent, and the effect observed in the 5% PPEE-SLNs cream group (G5) was comparable to the result in the positive control group (G3), which was treated with a market product (Figure 6) and showed significantly better anti-wrinkle scores compared with standard anti-wrinkle cream. The photographs are presented in Figure 7. Groups (G3–G5) showed very smooth and improved skin surfaces. There were no skin changes in G1 animals. In contrast, G2 showed thick and deep wrinkles.

#### 3.5.2. Histological Evaluation

**Figure 8:**
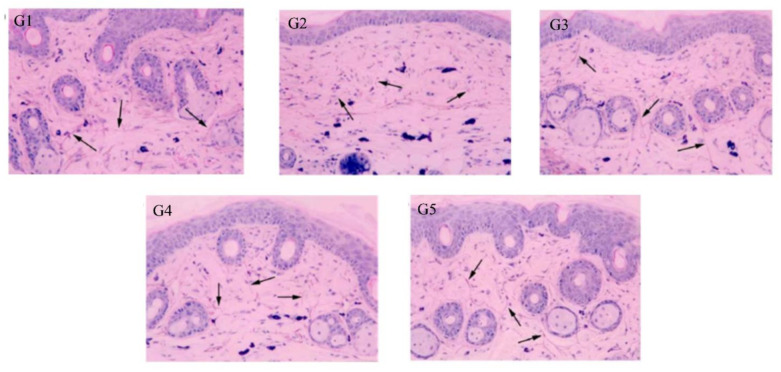
Histological observation of mice skin stained with hematoxylin-eosin. **G2** showed a significant decline in elastic fibers compared to **G1**. Treated groups had enhanced elasticity compared to **G2**. All treated groups, G3-G5 showed enhanced elasticity. The arrow represents elastic fibers viewed at ×200 magnification.

**Figure 9:**
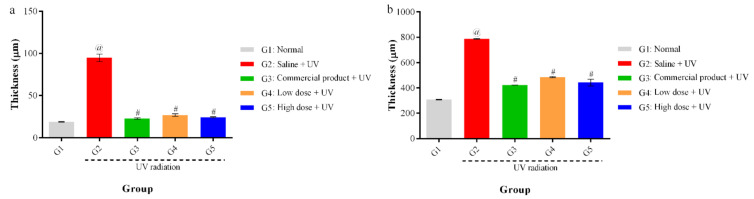
Effect of treatment on the thickness of (**a**) epidermis, (**b**) dermis. @: significant difference in comparison to normal group #: significant difference in comparison to control group *p* < 0.05/*n* = 10.

The elastic fibers were significantly decreased by UV irradiation compared with those of the normal group. Skin images for untreated and treated mice were observed by microscope (Figure 8). G1 had normal skin thickness of both dermis and epidermis and contained fibroblasts, in addition to, normal elastic fibers thickness with no fragmentation. Compared with G1, skin from the UV-irradiated mice (G2) showed a significant increase in thickening of both epidermis and dermis as well as a decrease in the formation of fibroblast and the elastic fibers by UV irradiation. Topical application of 5% (G5) and 2% (G4) PPEE-SLNs cream were comparable to that of G3 that received the commercial product. All G4 and G5 significantly decreased the thickness of dermis and epidermis, increase the content of fibroblasts as its number determines the content of collagen fibers, thus helping to repair damaged skin and reducing the aging effect on the skin (Figure 9a,b). Also, both groups showed preventive effects against the degradation of elastic fibers by UV irradiation, so we can conclude that treatment with either doses of PPEE-SLNs cream showed a protective effect against UV irradiation.

#### 3.5.3. Estimation of Superoxide Dismutase (SOD)

**Figure 10:**
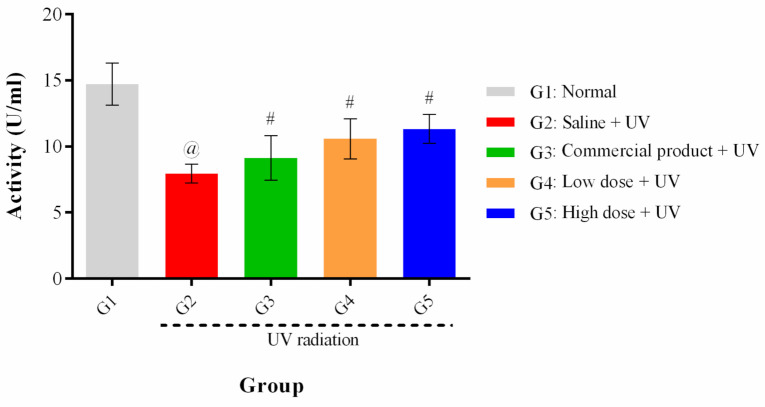
Effect of treatment on the SOD activity. All treated groups showed higher SOD activity compared to the G2 group with no significant difference among them. Data represented as the mean ± SD. @: significant difference in comparison to normal group #: significant difference in comparison to control group *p* < 0.05/*n* = 10.

To investigate the protective effects of PPEE-SLNs cream formulations on SOD activity, the SOD activity values of the normal (G1) and treatment groups (G3–G5) were compared with that of G2 (control group). The normal level of SOD activity was measured as 14.71 ± 1.58 U/mL, 184.79% higher than that of the normal group (7.96 ± 0.72 U/mL), which means that the SOD activity of the normal group was decreased by UV irradiation. The SOD activities of G5, G4, and G3 were 142.21%, 132.78%, and 114.57%, respectively, of that of the normal group, indicating that SOD activity was protected by the PPEE-SLNs cream formulations. Although no statistical differences in SOD activity were found among the treatment groups (G3–G5), the protective effect of (G5 and G4) against SOD reduction by UV irradiation was superior to that of the commercial product (G3) (Figure 10).

## 4. Discussion

Skin diseases present a significant health concern worldwide. They vary greatly in symptoms and severity and can be temporary or chronic. Among the most common is acne, the most common chronic skin inflammation [51], skin wrinkles directly linked to ECM degradation and skin pigmentation. Although these diseases’ pathology involves many factors, several studies indicate that oxidative stress is one of their major factors [52]. Oxidative stress can initiate inflammation and cause damage to cellular structures. However, it should be noted that in acne, oxidative stress may not be the sole cause. Bacterial infection and colonization play an additionally significant role in its pathogenesis through lipid peroxidation [53]. This highlights oxidative stress as a potential target for skin disease treatment by administrating both, local and systemic antioxidants.

Nowadays, although many techniques such as laser rejuvenation and synthetic products are available for the treatment of skin aging, the cosmetic industry is seeking alternative products of natural origin to avoid the hazards of synthetic ones. In this context, studies have focused on natural antioxidants as cosmeceutical ingredients that suppress UV-induced ROS, inhibit skin-related enzymes, and decrease the formation of melanin as an alternative to current treatment for the development of anti-aging skin care products.

One of the most important phytoconstituents in medicinal plants are polyphenols, in particular flavonoids. Flavonoids are a class of plant secondary metabolites with great cosmetic potential due to their excellent antioxidant, anti-inflammatory and antibacterial activities [54]. In addition, flavonoids have been suggested in treating the signs of aging by different mechanisms including their antioxidant properties by free radical scavenging and metal chelation with metalloenzymes providing antiprotease activities [55], sunscreen effect, and restoration of UV-induced DNA damage [56]. Genistein, myricetin, apigenin which are present in many fruits, herbs, and vegetables, proanthocyanidins, from grapes seeds, quercetin, and kaempferol in green tea have been reported to attenuate side effects caused by UV radiation [56,57,58]. Catechin, hesperidin, myricetin, rutin, quercetin have antioxidant and antiprotease activity that are beneficial in preventing skin aging [57].

Egypt occupies the tenth position globally in the production of peaches and nectarines, producing about 358,012 tons in 2019 [59]. *Prunus persica* (L.) var. Florida Prince is one of the most common peach varieties cultivated widely in Egypt. A previous study on other varieties of PP leaves by-products presents its use in food products, nutraceutical supplements, and as a cosmetic ingredient and emphasizing its high flavonoidal content [33]. On the other hand, given the cosmetic potential found in flavonoids and the reported potent antioxidant activities of PP leaves due to its high flavonoidal content. Hence, PP leaves have been chosen to evaluate their anti-wrinkle and skin whitening cosmetic potential as agricultural by-products. No previous studies were reported on the in-vitro antioxidants and skin-related enzymes’ activities of PPEE and to date, no anti-aging skincare preparations based on leaves by-products of PP var. Florida Prince using loaded SLNs exist to our knowledge.

In the present study, phenolic profiling of PPEE resulted in the isolation of an acylated flavonol glycoside with a rare structure, kaempferol 3*-O*-*β*-*^4^C_1_*-(6″-*O*-3,4-dihydroxyphenylacetyl glucopyranoside) KDPAG with high total phenolic and flavonoids content. There have been several studies proving that leaf extracts have a higher concentration of phenolic compounds than other parts of the same plant [14]. The in-vitro cytotoxicity evaluation showed the non-toxicity of PPEE, PPEE-SLNs due to the high percentage of cell viability. Extract-free-SLNs showed the highest percentage of cell viability as SLNs are composed of physiologically biocompatible and biodegradable lipids similar to lipid molecules of skin and thus, are safe carriers with high occlusion effect achieved without the use of paraffin and other greasy oils [60].

Powerful antioxidant properties of polyphenols were noted due to their redox activity, allowing them to serve as hydrogen donors, free radicals scavenging as well as their capacity to chelate metals [55]. Therefore, many methods were used to estimate the antioxidant properties in this study. Significant antioxidant capacities of PPEE against DPPH, ABTS and *β*-carotene assays compared to their respective standards. Potent antioxidant activities were showed by KDPAG using the same assays. *β*-carotene assay on PP leaves was first to be reported. Many studies reported that the *β*-carotene bleaching activity is linked to flavonoids which can inhibit oxidation of linoleic acid and the formation of hydroperoxides [14]. Acylated flavonoids, the class of KDPAG have been previously reported to have strong antioxidant activities [36]. Generally, antioxidant values were found to be higher than the ones reported in the literature. Differences between used extraction protocols can explain this point. This study was carried out using an ethanol extract of PP leaves wherein cited papers, extraction was done using acetone or methanol [20,61].

In the literature, TPC and TFC were significantly correlated with the antioxidant activity of PPEE confirming that polyphenols present in PPEE were a potent antioxidative agent and that the radical scavenging activity of PPEE is highly dependent on the flavonoidal content, mainly flavonols in the extract which is the core of the new isolate. TPC (*p* < 0.001) (r = 0.93, 0.96, 0.95, for DPPH, ABTS, *β*-carotene bleaching test, respectively) and TFC (*p* < 0.001) (r = 0.98, 0.99, 0.98, for DPPH, ABTS, *β*-carotene bleaching test, respectively). The results were in line with previous studies [20].

Collagenase, elastase, and tyrosinase are crucial enzymes involved in skin aging. Inhibiting the three enzymes will increase the strength of the skin, improve elasticity, avoid the development of dark spots, and thereby prevent the formation of wrinkles. The inhibitory effect of enzymes is either due to the active principle or the synergistic effect of different components in PPEE. The in-vitro findings of enzymatic inhibition showed that PPEE, PPEE-SLNs, and KDPAG possessed promising anti-aging and skin whitening activity, with regards to inhibition of elastase, collagenase and tyrosinase enzymes, and all were first to be reported. It was reported that PP fruit, seed, flower, and other species showed inhibition of elastase, collagenase, and tyrosinase [28,30,31,32]. Besides, anti-tyrosinase activity has been reported for acylated flavonoids, the class KDPAG [62].

KDPAG showed the highest% of inhibition against the three enzymes followed by PPEE-SLNs. PPEE, PPEE-SLNs, and KDPAG exhibited very good anti-elastase inhibition activity of 86.12 ± 1.42, 89.02 ± 2.31%, and 89.15 ± 1.26% which was statistically lower than *N*-(Methoxysuccinyl)-Ala-Ala-Pro-Val-chloromethyl ketone (91.12 ± 2.45%) (*p* < 0.01). In comparison, PPEE-SLNs and KDPAG showed anti-collagenase and anti-tyrosinase inhibition activities that were statistically higher (*p* < 0.01) than those of their positive controls (EDTA and kojic acid, respectively). On the other hand, PPEE showed similar (*p* > 0.05) collagenase inhibition to EDTA.

Furthermore, strong significant positive correlations were observed between TPC, TFC content of PPEE and the elastase, collagenase and tyrosinase inhibition (*p* < 0.001) (r = 0.841 and r = 0.893, respectively) for elastase inhibition, (*p* < 0.001) (r = 0.985 and r = 0.987, respectively) for collagenase inhibition and (*p* < 0.001) (r = 0.959 and r = 0.968, respectively) for tyrosinase inhibition. This suggests that phenolics and flavonoids may be the key components responsible for the inhibitory activity of PPEE.

In this study, the anti-collagenase activity may be due to the interaction of polyphenol hydroxyl groups with the backbone or other functional group side chain of collagenase or the hydrophobic interaction between the benzene ring of polyphenol and collagenase. These interactions result in conformational changes of the enzyme [63]. Moreover, flavonoids, the class of the newly isolated compound are known to be metal chelators by its 3-hydroxyflavon structure and bind to a Zn ion in the collagenase active site [64]. Also, the anti-tyrosinase activity can be explained by the binding of the hydroxyl groups of polyphenols through hydrogen bonding at the active site of the tyrosinase enzyme, leading to its inhibition [65]. Regarding elastase, hydroxyl groups of polyphenol and flavonoids forming bonds with the serine carboxyl groups at the elastase active site results in a non-functional enzyme [66]. In general, flavonoid-metal complexes with metalloenzymes have shown the potential to be SOD mimetics [67]. Chrysin, naringin, quercetin, and kaempferol, the core of KDPAG showed tyrosinase inhibitory effects [68]. Flavonols, the class of our new isolate, kaempferol, quercetin, and myricetin were reported to possess anti-elastase and anti-collagenase activity [67,69]. Also, a previous study showed that flavonols are stronger inhibitors of collagenase than flavones and isoflavones, indicating that the C-3-hydroxyl group is critical for a higher inhibitory activity [69].

The potential of biologically active compounds to penetrate the skin is highly critical to ensure delivery to the target site. Encapsulation techniques are primarily used to stabilize the easily reducible polyphenolics during storage and processing, thereby enabling their cosmetic and topical uses with enhanced antioxidant effects, dermal absorption, and penetration [70]. SLNs were prepared, characterized, and evaluated for its in-vitro skin permeability then formulated into anti-aging cream using two different concentrations (2% and 5%). Both cream formulae showed extended-release of PPEE over 24 h. The evaluation tests carried out for the formulated PPEE-SLNs anti-aging cream (2% and 5%) showed that PP leaves by-products are safe to be used in topical skin preparation to protect from intrinsic and extrinsic aging. The hypothesized mechanism of the anti-wrinkle activity of PPEE-SLNs cream can be explained as follows; the nano-formula reached the dermal layer where the antioxidant constituents have to be delivered and the penetration enhanced by the hydrating effect on the skin surface.

In-vivo anti-wrinkle activities of topically applied PPEE-SLNs (2% and 5%) were evaluated against UV-induced photoaging in a mice model using a wrinkle scoring method, tissue biomarkers (SOD), and histopathology. Either high or low dose PPEE-SLNs cream improves the appearance of wrinkles, decreased the thickness of dermis and epidermis, increases collagen content, prevents degradation of elastic fibers offering a highly significant protective effect against UV. Besides, the elevation of the detected antioxidant activity reflects the ability of PPEE-SLNs cream to significantly elevate SOD which goes on the same line with different studies that suggested the same protection against UV radiation [3]. PP leaves by-products are a potent natural antioxidant for combating skin aging.

Besides, depending on polyphenols’ mentioned properties constituting the main potential mechanisms of action against various skin disorders. Considering the increased bacterial resistance during the treatment of some skin disorders such as acne, plants’ phytoconstituents with high antioxidant and antimicrobial activity can increasingly be used as cosmetics therapeutics ingredients [51,71]. In this context, phenolic compounds and other antioxidants in PPEE leaves are valuable therapeutic ingredients with antioxidant and antimicrobial properties in preparations applied to the skin.

## 5. Conclusions

This is the first study to investigate the leaves by-products of PP var. Florida Prince for its potential as a cosmeceutical. PPEE was found to possess promising anti-aging activities by its capacities to inhibit DPPH, ABST, *β*-carotene oxidation, elastase, collagenase, and tyrosinase which may be correlated to its high phenolic and flavonoid content. Also, the isolation and structural elucidation of the unique acylated flavonol glycoside, KDPAG has not been previously reported. This compound is of important interest because it represents the first acylation with 3,4-dihydrocyphenyl acetic acid in association with flavonoid chemistry. The in-vitro cytotoxicity evaluation showed nontoxicity of PPEE and the optimized PPEE-SLNs. The in-vivo elastin expression and SOD activity results have shown that PPEE-SLNs formulations significantly protected wrinkle formation by UV irradiation. Based on the results obtained, this study recommended SLNs as novel carriers for dermal delivery of PPEE as it proved its potential to incorporate a natural antioxidant extract with high stability with no irritant effect on skin and hence enhance the performance as a cosmetic ingredient against skin aging and further recommended the study of the potential use of SLNs incorporated polyphenolics to overcome bacterial resistance problems as in acne through its ability to deliver such compounds. Finally, all the findings gave the evidence that PP leaves represent a good source of natural antioxidants and maybe a lead for the development of an innovative natural cosmeceutical with skin whitening and anti-wrinkle effects using agricultural by-products as starting raw material as well as using a novel delivery system. Also, it can represent a waste management solution for the agricultural food sector. In conclusion, the novel PPEE-SLNs formulations containing PPEE leaves by-products are a good candidate for topical PPEE delivery and useful for the development of anti-wrinkle preparations.

## Figures and Tables

**Table 1:** Formulation of PPEE-SLNs cream.

Ingredients	F1	F2
Span 80	1.5%	1.5%
Cetearath-20	3.5%	3.5%
Liq.Paraffin	7%	7%
Beeswax	2%	2%
Cetostearyl alcohol	6%	6%
Biocrol ws2	1%	1%
SLN	2%	5%
Propylene glycol	3%	3%
Purified water	74%	71%

**Table 2:** The different groups of the experiment. Treatment was done three times weekly for 40 days.

Group	Treatment
G1	Normal; saline treatment, not irradiated with UV
G2	Negative control; saline treatment, irradiated with UV
G3	Positive control; treated with a commercial cream (La Roche-Posay ^TM^ Active Vitamin C 10% Dermatological Anti-wrinkle Concentrate Intensive), irradiated with UV
G4	Treated with 2% PPEE-SLNs cream (low dose), irradiated with UV
G5	Treated with 5% PPEE-SLNs cream (high dose), irradiated with UV

**Table 3:** Effect of 300 μg/mL tested samples on the% inhibition along with the IC_50_ (μg/mL) of the different skin-related enzymes relative to their respective positive controls.

	Anti-Elastase		Anti-Collagenase		Anti-Tyrosinase	
	% Inhibition	IC_50_	% Inhibition	IC_50_	% Inhibition	IC_50_
**PPEE**	86.12 ± 1.42	55.88 ± 2.16	80.61 ± 1.28	311.91 ± 3.19	65.03 ± 1.85	362.5 ± 1.95
**PPEE-SLNs**	89.02 ± 2.31	54.01 ± 2.16	87.03 ± 1.31	283.92 ± 4.33	78.12 ± 1.50	303.03 ± 4.15
**KDPAG**	89.15 ± 1.26	49.80 ± 2.9	91.12 ± 2.45	267.66 ± 3.5	85.74 ± 2.10	290.19 ± 5.09
**Respective positive control**	92.52 ± 4.63	44.92 ± 1.71	79.82 ± 2.63	315.12 ± 2.21	76.52 ± 0.83%	321.65 ± 3.51

## Data Availability

Data is contained within the article.
